# A Comprehensive Self-Medication Management System to Reduce Medication Errors Among People Living With Dementia: Mixed Methods Feasibility and Usability Study

**DOI:** 10.2196/86828

**Published:** 2026-05-22

**Authors:** Santosh Basapur, Charles Gellman, Jamie B Plenge, Amelia Troutman, Erin Yurko, Brandon Woolsey, Justin McClendon, Lisa Marceau, Neelum T Aggarwal

**Affiliations:** 1 Department of Family and Preventive Medicine Rush University Medical Center Chicago, IL United States; 2 HiDO Technologies Folsom, CA United States; 3 Rush Alzheimer’s Disease Center Rush University Medical Center Chicago, IL United States; 4 Department of Neurological Sciences Rush University Medical Center Chicago, IL United States

**Keywords:** dementia, Alzheimer disease, medication adherence, self-medication device, caregiver burden, digital health, robotics, remote monitoring

## Abstract

**Background:**

Medication adherence is a critical challenge for people living with dementia and their caregivers. Standard care relies on appropriate medication management, yet there are few effective options beyond manual pill-counting and caregiver-administered dosing. These methods are prone to errors and impose a significant burden. Technologically enhanced adherence tools include smart caps, reminder apps, and electronic dispensers, which improve tracking but still depend on manual interaction, lack clinical integration, and are often unsuitable for cognitive impairment. The HiDO HomeCare System (HCS) is an artificial intelligence–enabled self-medication device, advancing the field by removing manual pill counting, automating the chain-of-custody, verifying consumption, and logging medication adherence through neuroscience-based logic and real-time monitoring.

**Objective:**

This study evaluated the feasibility, usability, and performance of the HCS for at-home medication management in dyads of people living with dementia and their caregivers. We examined setup, dispensing accuracy, task efficiency, and user satisfaction.

**Methods:**

A pooled analysis usability study was conducted with 35 caregiver-patient dyads at Rush University Medical Center, combining 2 sequential in-clinic cohorts run at different time points with the same protocol. Participants were recruited from the Rush Memory Clinic and Rush Alzheimer’s Disease Center data repository. Participant dyads completed device setup, medication dispensing, and simulated medication use with the system’s automated logging and dual-camera verification. Dyads repeated dispensing and simulated medication use following automated reminders sent to their mobile device. Dyads were encouraged to repeat dispensing tasks multiple times. Quantitative measures included setup time, time to dispense, success rates, reliability, and System Usability Scale scores. Qualitative measures also captured caregiver perceptions of usability, acceptability, and burden.

**Results:**

All 35 dyads successfully completed at least one dispensing task using the HCS. The average time for the first dispense attempt was 1:41 (SD 1:13) minutes (n=35). The second attempt averaged 1:35 (SD 1:10) minutes (n=21). Attempt 3 averaged 2:03 (SD 1:34) minutes (n=6). The system maintained accuracy across all users, with some variability in timing across age groups. The HCS received an overall mean System Usability Scale score of 70.2 (SD 18.2; n=34), reflecting above-average usability of the device. Caregivers reported that access to a system like HCS could reduce stress associated with medication administration and recommended improvements to specific design elements.

**Conclusions:**

The HCS demonstrated early feasibility, accuracy, and usability as a self-medication device tailored to people living with dementia. By automating the medication safety chain from delivery through consumption, HCS reduces caregiver workload and enhances patient safety and medication management. These findings support HCS as a viable medication adherence solution, addressing limitations of prior devices and enabling better dementia care. Larger-scale, longitudinal studies are planned to examine clinical outcomes, caregiver burden, and cost-effectiveness within real-world home and community settings.

## Introduction

### Medication Management Challenges

By 2060, the number of individuals living with Alzheimer disease and Alzheimer disease–related dementias in the United States is projected to double [1]. People living with dementia-related conditions currently have no known cure despite ongoing research, leaving patients primarily reliant on medication-based treatments to manage symptoms and preserve quality of life. People living with dementia have quality of life needs similar to those without dementia, including the ability to complete activities of daily living. Preserving these functions is critical to support independence, enable individuals to remain at home longer, and reduce both caregiver burden and cost of care [2]. Because medication is the primary treatment pathway, the need for appropriate, low-burden medication management is central to maintaining both cognitive and physical health [3]. This can be particularly challenging, as people living with dementia often have multiple chronic conditions such as cardiovascular disease, diabetes, and hypertension [4]. Polypharmacy and cognitive decline are strongly associated with morbidity, higher health care costs, and earlier institutionalization [3,5]. Inadequate management of medications for these conditions accelerates functional decline, increases avoidable hospitalizations, and shortens time at home.

Medication mismanagement is defined as missed doses, incorrect timing, or taking the wrong drug, and is a common and escalating challenge among people living with dementia [3]. As dementia progresses and patients struggle to manage both dementia-related medications and treatments for comorbid conditions, mismanagement leads to disrupted sleep, heightened anxiety, poor nutrition, and increased depression [6,7]. This, in turn, fuels a cycle of decline that often results in institutionalization. These challenges also contribute to caregiver burden, manifesting as stress, depression, and health deterioration that further increase risk for emergency visits and hospitalizations of caregivers, which increases risks to people living with dementia [3,8,9].

Research has established that stabilizing underlying chronic conditions is critical for preserving brain health in people living with dementia, and the most common comorbidities are associated with impaired cerebral blood flow and oxygen delivery, which in turn contribute to neuroinflammation and accelerated decline [10]. Thus, adherence to consistent medication use and ongoing monitoring can impact disease progression and stabilize cognition [11].

Medication mismanagement has far-reaching economic consequences. Hospital stays cost Medicare nearly 3 times more for dementia patients than for other older adults, and these hospitalizations often lead to long-term care facility admissions rather than a return home [1,12]. The average cost of a nursing home stay for dementia care is estimated at US $28,078 per patient every day a patient is prematurely removed from the home, which adds to the billions of dollars per year spent on caring for people living with dementia [13]. Over 11 million individuals provide unpaid care to people living with dementia, resulting in over 18 billion hours of care valued at nearly US $350 billion annually [1,14]. Premature institutionalization due to medication errors represents a modifiable factor that amplifies both direct health care expenditures and the emotional and financial burden on families.

### Scientific Premise

Memory relies on distinct systems in the brain. Declarative memory, mediated by the hippocampus and related temporal lobe structures, supports conscious recall and is severely impaired in Alzheimer disease [15]. Procedural (habit) memory is managed in the neostriatum and often remains intact for patients with Alzheimer disease [16]. Preserved habit memory in this population supports unconscious, repetitive learning, allowing people living with dementia to maintain routine tasks such as brushing teeth, dressing, or taking medication, even when declarative memory fails. Recognizing and building on this distinction provides an evidence-based rationale for habit-centered interventions to improve medication management.

### The HomeCare System

The HomeCare System (HCS) is an innovative self-medication device (SMD) that builds upon this neuroscience-informed foundation. Current models of SMDs rely heavily on user programming and frequent manual loading, which are contraindicated for patients with dementia. The HCS device reduces common home medication errors by automating safeguards to ensure that the right patient receives the correct medication at the correct dose, limiting manual aspects of medication management. The design reduces reliance on manual activities and leverages habit-forming memory systems through chaining logic, a behavioral method that breaks down complex routines into manageable, repeatable steps. This approach fosters habit formation and supports independence without requiring explicit recall. Subsequent dispensing and monitoring activities are conducted by the system. The device is designed to reduce errors and relieve caregiver burden by automating the medication safety chain from delivery to consumption. Core functions include (1) encoded medication data into RFID-enabled caps with automated detection of dosage and scheduling for up to 90-day supplies, (2) automated dispensing with chain-of-custody verification and logging, (3) dual-camera FaceID authentication, (4) clinical decision support for early intervention, and (5) customizable alerts and notifications for caregivers and providers delivered via mobile or electronic platforms. Real-time adherence monitoring is coupled with secure video capture of ingestion events, allowing for passive data collection on medication safety and user interactions.

The purpose of this study was to evaluate whether HCS features met the usability needs of patient-caregiver dyads. This was assessed by (1) in-clinic usability testing device set-up time less than 5 minutes, (2) completion of at least 75% of all usability testing tasks, (3) occurrence of no more than one noncritical error per dyad, and (4) mean System Usability Scale (SUS) score greater than 68. The secondary objective was to identify novel platform enhancements that could improve future device use in this population through qualitative feedback. Qualitative feedback was captured through group discussions with patient-caregiver dyads.

## Methods

### Study Design

HiDO Technologies, in collaboration with Rush University, conducted a human factors usability analysis study to validate the HCS prototype in dyads with people living with dementia and their caregivers. Two sequential in-clinic usability cohorts were run at different time points with the same protocol. Participants were recruited from the Rush Memory Clinic and Rush Alzheimer’s Disease Center data repository. For the analysis presented here, data were combined into a pooled dataset, enabling integrated evaluation of usability outcomes and replication of findings. The study was conducted in-clinic and focused on evaluating the system’s effectiveness in recognizing and accurately recording medication behaviors among persons with Alzheimer disease and related dementias.

### Study Population and Recruitment Process

The study was conducted by the Rush Alzheimer’s Disease Center at Rush University Medical Center, Chicago, Illinois. Participants were identified and recruited from 2 sources: the Rush Memory Clinic and the Rush Memory Clinic Data Repository. The Rush Memory Clinic, part of Rush University Medical Center and affiliated with the Rush Alzheimer’s Disease Center, is a referral clinic that offers comprehensive clinical evaluations for complaints of memory loss or other cognitive and behavioral changes, and recommendations for managing these conditions. The Rush Memory Clinic Data Repository is a center-maintained institutional review board (IRB)–approved registry that collects and stores information that may help answer future questions about Alzheimer disease and other diseases and conditions associated with memory and aging. Registry participants are given the opportunity to indicate their willingness to be contacted about current and future research studies affiliated with the Rush Alzheimer’s Disease Center.

Patients receiving clinical services at the Rush Memory Clinic and meeting preliminary inclusion criteria were identified and provided with information about the study (eg, IRB-approved study flyer) at the time of their clinical appointment or by phone afterwards. Registry participants meeting preliminary inclusion criteria who had indicated willingness to be contacted about future research studies were identified and contacted by phone by research staff to share study information and assess interest. All registry screening and study recruitment were carried out by research assistants at the Rush Alzheimer’s Disease Center, specifically trained in cognitive research protocols.

Interested individuals were asked a set of screening questions to confirm eligibility, such as age and availability of a caregiver (age 18 years or older) willing to participate. Individuals with cognitive deficits and/or serious mental health or medical conditions that may have compromised safety or the ability to provide accurate user feedback were excluded.

Eligible and interested individuals were scheduled for an in-clinic visit to obtain informed consent and participate in the usability testing and group discussion session. A study-specific informed consent process was conducted using IRB-approved consent forms. Staff explained study aims, procedures, risks, and assessments, and used teach-back methods to confirm understanding. Written documentation of informed consent was obtained from both dyad members. All data were collected under the direction of the investigators.

### Study Procedures

Once consented, dyads participated in usability testing and a group discussion session that included tasks related to the use of the HCS. HiDO staff, our human design expert (SB), and Rush Research Assistant team members gave a live demonstration of the HCS product and answered questions on device setup and medication dispensing prior to testing. There were 2 usability testing steps: setup and use. For the setup tasks, dyads were asked to conduct the HCS device setup, including completing facial recognition tasks and entering a security pin. The patients were required to complete this step, but were informed that if they needed assistance, they could consult their caregiver. Dyads were provided an opportunity to complete the setup up to 3 times. Repeated attempts were optional within the study visit, and lower numbers reflected participant preference rather than dropout after device failure.

Then, the patient was asked to use the HCS device to conduct a medication dispensing activity (vitamin C). The next activity was to successfully simulate “taking of medication” during the usability test, with video capture of this process. Taking medication was performed as a simulation, and no medication was actively taken. During device setup, dyads were asked to perform all device setup and registration steps using a “think-aloud” protocol. In a “think-aloud” protocol, the user completes each step in the process while saying it out loud. Participants were asked to say what came to their minds during the task. The think-aloud process was recorded on paper data forms by the research assistants during the activity.

Once the device was enabled according to the protocol, patients were asked to complete a second set of predefined device use tasks: (1) recognize the medication reminder on the HCS device and the mobile phone that was in the room with the patient—each dyad had a mobile phone paired to the HCS, (2) unlock the HCS device with facial recognition, and (3) successfully simulate consumption of the dispensed medication (vitamin C). As with setup, this was a simulation, and no medication was taken.

The study team recorded critical and noncritical errors (predefined by the study protocol as developed by the HiDO team and the investigators at Rush) encountered by the participants after the 2-part usability study was completed with each dyad. A critical error was defined as an error that prevented full deployment of the system, preventing medication from being dispensed or dispensed correctly. A noncritical error was defined as an error that diverted the participant from the intended path but did not significantly affect the usability of the system.

Following the setup and use testing, dyads were asked to complete an SUS score with a 0-to-100 rating to measure the perceived usability of the system, as well as a 7-point likeability scale on their preferences related to the HCS. After SUS and likeability ratings were captured, dyads participated in semistructured group discussions. Group discussions included feedback about the device usability, relevance to use in the everyday home environment, enthusiasm, perceptions on setup, medication delivery experience, device ergonomics, alerting, challenges, and suggested improvements. The group discussion was conducted in English in a private room in the Rush Alzheimer’s Disease Center conference room by the investigator (SB) with research assistants. The audio of the discussion was recorded and transcribed for analysis. Each session was scheduled for 60 minutes.

### Data Collection

Time to set up the device, device task completion rate, number and type of critical and noncritical errors, and SUS scores were quantitatively measured. Setup time was documented in minutes and seconds, and successfully completed tasks were counted and numbered. SUS and likeability scores (reflecting questions targeting overall enthusiasm, setup, medication delivery, device ergonomics, alerting, and challenges) were recorded per dyad. Critical and noncritical errors in device setup and management were recorded, including when to take medication, retrieving the dispensed pill, taking the dispensed pill, and technical interactions with the device. Semistructured interviews were analyzed for targeted feedback about platform features. HCS video and other recorded data from the usability testing were captured and stored as audio files in the Microsoft Teams site of Rush Alzheimer's Disease Center for this study. Recorded think-aloud data and SUS scores were saved as Microsoft Word documents.

### Ethical Considerations

The study was approved by the IRB at Rush University Medical Center (protocol 21033001-IRB01). All patient and caregiver participants provided written informed consent. All data used in this study were deidentified. Participants were compensated US $75 per dyad member (US $150 per dyad). The investigators and study staff monitored safety via adverse event reporting defined in the IRB-approved study protocol. Risks were minimal to participants, as they interacted with a device under close supervision by the study team in a clinical environment. No actual medications were administered during the study; vitamin C was dispensed by the platform but not consumed. Video capture was limited to the simulated dispensing session, stored in a secure deidentified form for analysis, and used only for protocol-defined usability assessment.

### Statistical Analysis

The primary objective of this human factors usability study was to evaluate functional performance and user interaction with the HCS, not to test a clinical hypothesis. Power calculations were based on standards for usability testing rather than inferential statistical power. U.S. Food and Drug Administration human factors research guidelines suggest that a sample of 15-20 participants per user group is typically sufficient to identify at least 90% of usability issues within a defined task set [17]. Our pooled sample of 35 patient-caregiver dyads exceeded this benchmark and provided sufficient power to assess primary usability endpoints, including task success rate, time-on-task, and SUS scores.

The primary endpoint was the mean SUS score, with a prespecified benchmark of ≥68 indicating above-average usability. Secondary endpoints included setup time (<5 minutes), task completion rate (≥75%), and error rate (≤1 noncritical error per dyad). Given the iterative design of the HCS, 2 independent cohorts were analyzed to confirm the replicability of findings and assess potential improvements between study phases. The pooled analysis was used to ensure adequate power for quantitative comparisons while capturing qualitative insights across both groups.

### Qualitative Data Analysis

All qualitative data collected during the usability sessions were analyzed to complement quantitative findings and identify areas for device optimization. Audio recordings from the “think-aloud” usability exercises and semistructured postsession interviews were transcribed verbatim and verified for accuracy by the research team at Rush Alzheimer’s Disease Center.

A hybrid deductive-inductive approach was applied. Deductive codes were derived from the study’s predefined domains of interest, including setup experience, device navigation, perceived usefulness, and caregiver burden. Inductive codes captured emergent insights related to user experience, accessibility, and design improvements. A trained researcher (SB) independently coded all transcripts. Codes were grouped into categories and overarching themes reflecting participant perceptions of usability, acceptability, and integration within daily routines. Given the formative usability purpose of the study, transcripts were coded by the trained researcher (SB) and discussed with the broader study team, including research assistants, to refine themes. This single-coder approach is a limitation.

A total of 35 transcripts were analyzed, and 547 quotes were coded, yielding 119 open codes and 5 primary themes that captured user experience variability across cohorts and age groups. These included (1) innovation and perceived value; (2) ease of use and learning curve; (3) physical design and environmental fit; (4) cognitive and emotional support for users; and (5) trust, safety, and the role of caregivers.

## Results

### Evaluation Results

#### User Statistics

A total of 316 dyads were contacted for study participation. Of those, 125 declined participation, 79 did not meet study eligibility, and 76 remained pending at the time enrollment closed (eg, did not return contact attempts and were still considering participation). A total of 35 dyads (people living with dementia + caregiver = dyad) participated in the study. Demographics and cohort details are defined in Table 1. Cohort 1 included 15 participants, and cohort 2 included 20 participants. People living with dementia were aged 50 years or older and diagnosed with mild cognitive impairment or early-stage dementia (Montreal Cognitive Assessment score 18 to 24, inclusive, or equivalent cognitive assessment) as noted in the chart.

**Table 1:** Sociodemographic characteristics by cohort and role (people living with dementia and caregivers).

Characteristics			Cohort 1			Cohort 2				Combined totals		
			People living with dementia (n=15)	Caregiver (n=15)		People living with dementia (n=20)		Caregiver (n=20)		People living with dementia (n=35)		Caregiver (n=35)
Age (years), mean (range)			72 (64-86)	64 (34-75)		75 (66-82)		69 (46-81)		74 (64-86)		67 (34-81)
**Sex, n (%)**												
	Female	11 (73)		9 (60)	10 (50)		12 (60)		21 (60)		21 (60)	
	Male	4 (27)		6 (40)	10 (50)		8 (40)		14 (40)		14 (40)	
**Race, n (%)**												
	Black or African American	4 (27)		5 (33)	3 (15)		4 (20)		7 (20)		9 (26)	
	More than one race, Black or African American, White Asian	1 (7)		0 (0)	0 (0)		0 (0)		1 (3)		0 (0)	
	Prefer not to answer	0 (0)		0 (0)	1 (5)		0 (0)		1 (3)		0 (0)	
	White	10 (67)		10 (67)	16 (80)		16 (80)		26 (74)		26 (74)	
**Level of education, n (%)**												
	Associate's degree	1 (7)		1 (7)	0 (0)		0 (0)		1 (3)		1 (3)	
	Bachelor's degree or higher	11 (73)		11 (73)	13 (65)		17 (85)		24 (69)		28 (80)	
	Primary level education	0 (0)		0 (0)	0 (0)		1 (5)		0 (0)		1 (3)	
	Secondary level education, GED^a^, or equivalent	1 (7)		1 (7)	2 (10)		1 (5)		3 (9)		2 (6)	
	Some college	2 (13)		2 (13)	4 (20)		1 (5)		6 (17)		3 (9)	
	Trade/vocational training	0 (0)		0 (0)	1 (5)		0 (0)		1 (3)		0 (0)	
**Marital status, n (%)**												
	Divorced	0 (0)		2 (13)	2 (10)		3 (15)		2 (6)		5 (14)	
	Married	12 (80)		12 (80)	16 (80)		16 (80)		28 (80)		28 (80)	
	Never married	1 (7)		1 (7)	1 (5)		1 (5)		2 (6)		2 (6)	
	Widowed	2 (13)		0 (0)	1 (5)		0 (0)		3 (9)		0 (0)	

^a^GED: General Education Development.

Across both cohorts, the overall mean age of patients was 74 (range 64-86) years and caregivers averaged 67 (range 34-81) years. In cohort 1, people living with dementia averaged 72 (range 64-86) years and caregivers averaged 64 (range 34-75) years; in cohort 2, people living with dementia averaged 75 (range 66-82) years and caregivers averaged 69 (range 46-81) years. Overall, 60% (21/35) of the people living with dementia were female. By race, 74% (26/35) were White, 20% (7/35) Black or African American, 3% (1/35) reported more than one race, and 3% (1/35) preferred not to answer. The level of education was predominantly higher, with 69% (24/35) holding a bachelor’s degree or higher, 17% (6/35) reporting some college, 9% (3/35) reporting secondary level education or General Education Development. Most participants were married (28/35, 80%), with 6% (2/35) divorced, 6% (2/35) never married, and 9% (3/35) widowed. Patterns were similar across cohorts.

#### Setup Times

Across the key measures, the overall average initial setup time was 2:59 minutes (n=35). Setup time varied by cohort 1 (3:31 minutes) and cohort 2 (2:35 minutes), with cohort 2 requiring slightly less time than cohort 1 across age groups. See Tables 2 and 3.

**Table 2:** System usability score, setup, and task attempt completion by phase and age^a^.

Cohort and age (years)			Participants, n		SUS^b^, mean (SD)		Setup 1 (min:sec), mean (SD)		Setup 2 (min:sec), mean (SD)	Attempt 1 (min:sec), mean (SD)		Attempt 2 (min:sec), mean (SD)
**Cohort 1**												
	64-69	7		83.6 (15.1) (n=7)		02:55 (01:13) (n=7)		N/A^c^ (n=0)		00:57 (00:29) (n=7)	01:26 (00:25) (n=2)	
	70-74	2		73.8 (12.4) (n=2)		03:44 (00:23) (n=2)		N/A (n=0)		03:25 (02:50) (n=2)	03:22 (N/A) (n=1)	
	75+	6		75.4 (23.9) (n=6)		04:08 (01:51) (n=6)		02:18 (00:04) (n=2)		02:31 (00:58) (n=6)	01:48 (01:37) (n=5)	
	Overall	15		79 (18.2) (n=15)		03:31 (01:29) (n=15)		02:18 (00:04) (n=2)		01:54 (01:24) (n=15)	01:54 (01:23) (n=8)	
**Cohort 2**												
	64-69	4		66.7 (12.6) (n=3)^d^		02:47 (01:39) (n=4)		01:41 (00:24) (n=2)		01:54 (01:44) (n=4)	01:42 (01:50) (n=3)	
	70-74	4		61.2 (2.5) (n=4)		02:49 (00:30) (n=4)		N/A (n=0)		01:10 (00:30) (n=4)	00:53 (00:16) (n=4)	
	75+	12		63.1 (18.5) (n=12)		02:26 (00:47) (n=12)		N/A (n=0)		01:32 (00:59) (n=12)	01:32 (00:55) (n=6)	
	Overall (cohort 2)	20		63.3 (15.2) (n=19)^d^		02:35 (00:55) (n=20)		01:41 (00:24) (n=2)		01:32 (01:04) (n=20)	01:22 (01:02) (n=13)	
All (both cohorts)			35		70.2 (18.2) (n=34)^d^		02:59 (01:16) (n=35)		01:59 (00:25) (n=4)	01:41 (01:13) (n=35)		01:35 (01:10) (n=21)

^a^Each cell includes the number of participants for that metric in that subgroup.

^b^SUS: System Usability Scale.

^c^N/A: not applicable.

^d^Indicates fewer SUS observations than the total number of participants per row. One participant had missing SUS scores.

**Table 3:** Overall system usability and task completion by age group^a^.

Age (years)	SUS^b^, mean (SD)	Setup 1 (min:sec), mean (SD)	Attempt 1 (min:sec), mean (SD)	Attempt 2 (min:sec), mean (SD)	Time improvement attempt 1>2 (min:sec)	Attempt 3 (min:sec), mean (SD)	Time improvement attempt 2>3 (min:sec)
64-69	78.5 (15.9) (n=10)	02:52 (01:18) (n=11)	01:18 (01:08) (n=11)	01:35 (01:19) (n=5)	+00:18 (n=5)	01:11 (N/A^c^:N/A), (n=1)	–00:24 (n=1)
70-74	65.4 (8.7) (n=6)	03:07 (00:38) (n=6)	01:55 (01:46) (n=6)	01:23 (01:08) (n=5)	–00:32 (n=5)	05:06 (N/A:N/A) (n=1)	+03:43 (n=1)
75+	67.2 (20.6) (n=18)	03:00 (01:27) (n=18)	01:52 (01:04) (n=18)	01:40 (01:13) (n=11)	–00:12 (n=11)	01:30 (00:34) (n=4)	–00:10 (n=4)
Overall	70.2 (18.2) (n=34)	02:59 (01:16) (n=35)	01:41 (01:13) (n=35)	01:35 (01:10) (n=21)	–00:07 (n=21)	02:03 (01:34) (n=6)	+00:28 (n=6)

^a^Each cell includes number of participants for that metric in that subgroup.

^b^SUS: System Usability Scale.

^c^N/A: not applicable.

#### Medication Reminder and Dispensation of Medicine

All participating dyads (n=35) successfully dispensed medications using the HCS (Tables 2 and 3). Participants were allowed to complete dispensing as many times as they desired, with attempts ranging from 1 to 5 across the sample. Each attempt required participants to respond to a reminder, unlock the device via FaceID, and dispense medications, with time intervals between steps ranging from 5 to 15 minutes from reminder to completion.

Across all 35 participants, the average time for the first dispense attempt was 1:41 minutes (n=35). The second attempt averaged 1:35 minutes (n=21). Attempt 3 was conducted in an average of 2:03 minutes (n=6). The 64-69-year-old age group completed the first attempt faster than the 70-74 or 75+ age groups. Time improvement between attempt 1 and attempt 2 improved overall by 0:07 seconds, with improvement for the 70-74-year-old (–0:32 seconds, n=5) and 75+ age group (–0:12 seconds, n=11), and +0:18 seconds (n=5) for the 64-69-year-old age group. Setup and attempt times between cohort 1 and cohort 2 varied, with overall times slightly faster in cohort 2.

#### SUS Ratings and Task Performance

The overall mean SUS score across all participants was 70.2 (SD 18.2; n=34) (Tables 2 and 3), reflecting an above-average usability score. Mean SUS scores differed between the two study cohorts. Cohort 1 participants reported a mean SUS score of 79.0 (SD 18.2; n=15), and cohort 2 participants reported a mean score of 63.3 (SD 15.2; n=19). In cohort 1, the 64-69 age group reported the highest score at 83.6 (SD 15.1; n=7), compared to 73.8 (SD 12.4; n=2) for 70-74-year-olds and 75.4 (SD 23.9; n=6) for the 75+ group. In cohort 2, the 64-69 group scored 66.7 (SD 12.6; n=4), the 70-74 group scored 61.2 (SD 2.5; n=4), and the 75+ group scored 63.1 (SD 18.5; n=12).

Analysis of the pooled data by age group is summarized in Tables 2 and 3. The 64-69-year-old age group overall reported the highest mean SUS rating at 78.5 (SD 15.9, n=10). This decreased to 65.4 (SD 8.7; n=6) for 70-74-year-olds and was 67.2 (SD 20.6; n=18) for the 75+ year-old cohort. Task performance data demonstrated that the overall mean time for the initial task attempt (attempt 1) was 01:41 (SD 1:13; n=35). The overall mean time for the subsequent attempt (attempt 2) was 01:35 (SD 1:10; n=21), resulting in an average time improvement of 7 seconds (–0:07; n=21). Time improvement varied by age group: the 70-74-year-old age group improved on average by 32 seconds (–0:32; n=5), while the 75+ year-old age group improved by 12 seconds (–0:12; n=11). The 64-69-year-old age group took an average of 18 seconds (0:18; n=5) on their second attempt.

#### Likert Scale Findings: Usability

**Figure 1:**
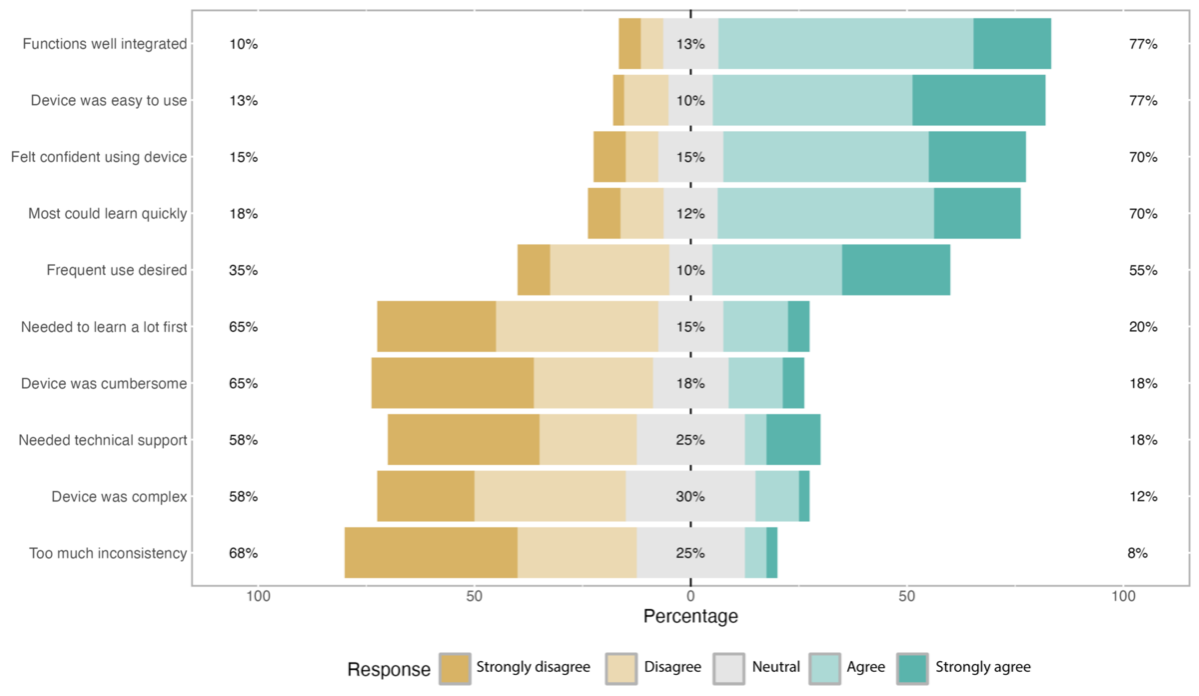
System Usability Scale scores on device usability.

Several findings were reported related to usability based on the SUS [17] (Figure 1). Most participants (27/34, 77%) agreed or strongly agreed that the device was easy to use, the device’s functions were well integrated (27/34, 77%), and reported confidence using the device (24/34, 70%). Over half of the participants agreed or strongly agreed that they needed technical support to use the device (20/34, 58%), the device was complex (20/34, 58%), and they needed to learn a lot before using the device (23/34, 65%).

#### Critical and Noncritical Errors

Analyses of participant errors of the pooled sample (n=35) are reported in Table 4. All errors were recorded, and more than one error could be attributed to one participant. Two critical errors were recorded in the pooled sample, both of which occurred in cohort 1 by two unique users. There were 17 noncritical errors recorded by 11 unique participants in the pooled sample. In cohort 1, eight participants made 14 noncritical errors, and in cohort 2, three participants made 3 noncritical errors. The 75+ age group demonstrated the highest frequency of noncritical errors, with 4 participants accounting for 8 total errors. Errors were tracked through the HCS video interface.

**Table 4:** Critical and noncritical errors by cohort and age group^a^.

Cohort and age (years)		Participants, n	Participants with ≥1 critical error^b^, n	Total critical errors^c^, n	Participants with ≥1 noncritical error^b^, n	Total noncritical errors^c^, n
**Cohort 1**						
	64-69	7	1	1	3	5
	70-74	2	0	0	1	1
	75+	6	1	1	4	8
	Overall	15	2	2	8	14
**Cohort 2**						
	64-69	4	0	0	1	1
	70-74	4	0	0	0	0
	75+	12	0	0	2	2
	Overall	20	0	0	3	3
Overall (both cohorts)		35	2	2	11	17

^a^The table presents (1) the number of unique participants with at least one critical or noncritical error and (2) the total number of errors recorded for each group. “Overall” rows summarize all age groups within each cohort; the bottom row pools both cohorts. Errors were scored based on predefined definitions and coded in the Participant In-Clinic Usability Case Report Form.

^b^Participants with ≥1 error: number of people who made at least one error of that type.

^c^Total errors: sum of all observed errors, including multiple errors by the same participant.

### Qualitative Results

#### Themes From Qualitative Data

Five primary themes emerged across the 35 dyad interviews which included (1) innovation and perceived value represented as enthusiasm for a technology that could reduce caregiver workload; (2) ease of use and learning curve, or the need for clearer setup instructions and guidance; (3) physical design and the environmental fit, accessibility, and ergonomics including preferences for larger fonts, higher contrast, and adjustable display angles; (4) cognitive and emotional support for users; and (5) trust, safety and the caregiver’s role and support for shared use reflecting the desire for connected tools and training resources. Themes are summarized with illustrative quotes from conversations.

#### Theme 1: Innovation and Perceived Value

Participants viewed the HiDO system as an innovative solution to improve medication adherence and caregiver support. They valued the automation and accountability features, which simplified routines or reduced caregiver burden: “great technology for someone with memory issues.” Some participants reflected satisfaction with pillboxes and found the device complex, while others felt it created accountability to take medications.

#### Theme 2: Ease of Use and Learning Curve

Participants described the device as “self-explanatory” and “easy once set up,” yet several admitted to feeling “overwhelmed at first” or “intimidated by all the steps.” Participants recommended simplifying the touchscreen interface with larger fonts, slower response time, and multimodal feedback (eg, auditory or visual cues). Most users achieved proficiency after repeated use, with one participant summarizing, “There’s a learning curve, but it’s simple once you get the hang of it.”

#### Theme 3: Physical Design and Environmental Fit

Participants consistently described the device as bulky and difficult to integrate into home environments. Size, need for continuous power, and aesthetic incongruence were perceived as barriers. Many suggested a smaller or wall-mounted design, enhanced portability, and customizable color or appearance to reduce stigma, with participants noting, “I’d put it in my kitchen if it looked more like an appliance.”

#### Theme 4: Cognitive and Emotional Support for Users

The device’s reminder and monitoring capabilities provided reassurance and supported autonomy for some users. Participants valued multimodal alerts and personalized prompts and suggested these could be enhanced. Emotional themes centered on feeling empowered and less dependent on caregivers, with one participant summarizing, “It helps you feel you’re still in control of your medications.” Usability was seen as optimal for mild cognitive impairment but potentially limited for users with advancing dementia and suggested progressive usability that evolves with cognitive decline.

#### Theme 5: Trust, Safety, and the Role of Caregivers

Facial recognition and dispensing safeguards enhanced perceived safety, but users also expressed apprehension about malfunction or data misuse. The presence of caregivers was viewed as critical, especially for setup, refilling, and troubleshooting. As one caregiver noted, “You can’t rely on the phone ping; you need something that tells me if they didn’t take it.” Participants highlighted the tension between independence and monitoring.

## Discussion

### Implication of Our Usability Evaluation

Our study assessed the usability and functionality of the HCS with 35 dyads, including setup and use capability, system usability, and qualitative feedback. Two cohorts with similar participants were included in a pooled analysis. On average, the overall time from reminder recognition to medication dispensation was 1.41 minutes (n=35), which was below the prespecified 5-minute threshold for device setup. Task performance data suggest the device was able to be set up and engaged with by the user population across age groups. There were noted differences by age and attempt, suggesting improvements when making repeated attempts, but the sample size was not designed for subgroup analysis. There were low instances of critical errors across the study, indicating the built-in safeguards were performing as designed. The noncritical errors (greater than or equal to 1) helped identify areas that need further work and modifications in product design, especially onboarding user experience and operating manual design.

The overall SUS ratings exceeded the threshold score of 68, typically considered above average, although scores varied between cohort 1 (79) and cohort 2 (63.3). Within age group variations are important to note for future research, but the sample size of 35 participant dyads was not designed to address subgroup analyses. The difference between the two cohorts may reflect iterative improvements in study implementation rather than inherent usability differences. Cohort 1 participants were the first users to engage in the HCS in this study, and likely had greater staff engagement to ensure the protocol was being followed and to address any unforeseen challenges. This could have contributed to slightly longer completion times in cohort 1. Cohort 2 may have benefited from refinements in user instructions and onboarding. Although both cohorts achieved above-average SUS scores for usability, the lower SUS scores in cohort 2 may also reflect slightly higher staff engagement with the setup and onboarding activities in cohort 1, which could have established a different rapport during the process than in cohort 2. SUS scores also represented valuable insights into usability for in-home SMDs with patients who are cognitively impaired. It adds value to the field that our data demonstrate an SMD like the HCS is reported as easy to learn and use, and participants felt confident using it, but further work is required to build in simple, clear, and precise operating and use instructions to meet the users’ needs.

Qualitatively, participants recognized the HCS’s potential to improve medication safety and reduce caregiver stress. Adoption was moderated by perceived complexity, physical design, and the degree of human connection integrated into the technology. Findings underscore that usability for populations with cognitive impairment requires iterative design emphasizing simplicity, adaptability, and trust.

Accounting for the between-cohort differences, findings from this study make a significant contribution to the role of SMDs in supporting the challenges of medication adherence in people with dementia and cognitive impairment. Medication adherence tools have undergone substantial transformation since the 1950s, evolving across 4 generations. Early solutions (Generation 1) relied entirely on manual methods such as pill bottles and organizers, placing the full responsibility on patients and caregivers. Generation 2 introduced digital aids, including smart caps and mobile apps, which provided reminders and basic tracking but remained limited by their dependence on user input. Generation 3 technologies advanced to scheduled dispensers with remote monitoring features, yet they remained insufficient for individuals with cognitive decline, particularly those with Alzheimer disease. Current Generation 4 solutions seek to alleviate user burden by automating manual tasks and incorporating advanced monitoring and intervention features.

### Principal Findings

The HCS represents a novel advancement within this fourth generation, extending usability to one of the most vulnerable patient groups—people living with dementia. This population faces unique challenges, including impaired cognitive recall, difficulties with complex device setup, and heavy reliance on caregivers. Findings from this study underscore the potential of the HCS to address these barriers. Participants emphasized the perceived usefulness of an automated medication management system, alleviating reliance on caregivers and reducing burden on patients by accessing habit memory. The automated reminders and facial recognition were noted as valuable for simplifying access, while participants also identified areas for improvement such as device size, display accessibility, and clearer setup support. These insights highlight both the feasibility and the refinement opportunities of a fully automated adherence system in real-world home settings.

### Strengths and Limitations

This study has a few important limitations. Recruitment focused on patients who were already being treated in a clinical care setting, which may have limited the diversity of the participants to the available demographics of the Rush Medical Center. While efforts were made to include a diverse sample, the population for both patient and caregiver was two-thirds White and one-third Black, without broader racial and ethnic representation. The participant cohort was more likely to be educated, with most participants holding a bachelor’s degree or higher. Because of these factors, our cohort may not represent all older adults taking multiple medications. However, as the primary outcome of this study was to demonstrate the feasibility of HCS to determine usability changes and enhancements in setup and use, a random and representative sample was not feasible or critical to achieve these initial goals. Two important strengths were noted. First, we conducted the study with patient and caregiver dyads in two separate but comparable cohorts, providing a larger sample and a broader dataset for analysis. Second, we incorporated qualitative methods, which enabled dyads to share valuable insights that could improve the HCS device for future implementation.

### Conclusions

Demonstrating feasible use in people living with dementia, the HCS has the potential to improve the standard of care for medication adherence more broadly. Unlike prior generations of adherence tools, the HCS integrates features that account for both cognitive and caregiving demands, addressing a critical innovation gap specifically in a patient population with limited options for automated medication dispensing. Addressing usability and functionality of the HCS with patient and caregiver dyads advances the potential for more effective medication management in people living with dementia. Results of this study provide the foundation for future studies to measure medication management compliance, impact on disease progression, prolonged quality of life, and more effective communication between patient, caregiver, and clinical care team.

## Data Availability

The datasets generated or analyzed during this study are available upon reasonable request and in accordance with data sharing policies and with respect for intellectual property and proprietary data constraints. A request for data can be placed by emailing the corresponding author.

## Abbreviations

HCS: HomeCare System
IRB: institutional review board
SMD: self-medication device
SUS: System Usability Scale
